# Comparison of Calcium Balancing Strategies During Hypothermic Acclimation of Tilapia (*Oreochromis mossambicus*) and Goldfish (*Carassius auratus*)

**DOI:** 10.3389/fphys.2018.01224

**Published:** 2018-09-03

**Authors:** Tsung-Yu Han, Chien-Yu Wu, Han-Chuan Tsai, Yi-Pei Cheng, Wei-Fan Chen, Tzu-Chien Lin, Chia-Yih Wang, Jay-Ron Lee, Pung-Pung Hwang, Fu-I Lu

**Affiliations:** ^1^Department of Biotechnology and Bioindustry Sciences, College of Bioscience and Biotechnology, National Cheng Kung University, Tainan, Taiwan; ^2^Department of Life Sciences, National Chung Hsing University, Taichung, Taiwan; ^3^Department of Cell Biology and Anatomy, College of Medicine, National Cheng Kung University, Tainan, Taiwan; ^4^Institute of Cellular and Organismic Biology, Academia Sinica, Taipei, Taiwan; ^5^The iEGG and Animal Biotechnology Center, National Chung Hsing University, Taichung, Taiwan

**Keywords:** Ca^2+^ influx, Ca^2+^-ATPase, cold acclimation, teleost, gill

## Abstract

The body temperatures of teleost species fluctuate following changes in the aquatic environment. As such, decreased water temperature lowers the rates of biochemical reactions and affects many physiological processes, including active transport-dependent ion absorption. Previous studies have focused on the impacts of low temperature on the plasma ion concentrations or membrane transporters in fishes. However, very few *in vivo* or organism-level studies have been performed to more thoroughly elucidate the process of acclimation to low temperatures. In the present study, we compared the strategies for cold acclimation between stenothermic tilapia and eurythermic goldfish. Whole-body calcium content was more prominently diminished in tilapia than in goldfish after long-term cold exposure. This difference can be attributed to alterations in the transportation parameters for Ca^2+^ influx, i.e., maximum velocity (*V_max_*) and binding affinity (1/*K_m_*). There was also a significant difference in the regulation of Ca^2+^ efflux between the two fishes. Transcript levels for Ca^2+^ related transporters, including the Na^+^/Ca^2+^ exchanger and epithelial Ca^2+^ channel, were similarly regulated in both fishes. However, upregulation of plasma membrane Ca^2+^ATPase expression was more pronounced in goldfish than in tilapia. In addition, enhanced Na^+^/K^+^-ATPase abundance, which provides the major driving force for ion absorption, was only detected in tilapia, while upregulated Na^+^/K^+^-ATPase activity was only detected in goldfish. Based on the results of the present study, we have found that goldfish and tilapia differentially regulate gill epithelial plasma membrane Ca^2+^-ATPase (PMCA) expression and Na^+^/K^+^-ATPase activity in response to cold environments. These regulatory differences are potentially linked to more effective regulation of Ca^2+^ influx kinetics and better maintenance of whole body calcium content in goldfish than in tilapia.

## Introduction

Most freshwater teleost species live and grow in environments with Ca^2+^ concentrations that are low compared to the concentrations in their body fluids. Despite this relatively low availability from the environment, constitutive increase of whole-body calcium content is an important factor during development that affects survival (Chou et al., 2002; Chen et al., 2003). Therefore, active uptake of Ca^2+^ is requisite for fish to live and grow in fresh water. Ca^2+^ uptake from the environment mainly occurs via transcellular transportation across the gill epithelium, and this process is dependent on facilitated diffusion through the epithelial Ca^2+^ channel (ECaC) on the apical membrane (Pan et al., 2005), active transport by PMCA and Na^+^/Ca^2+^ exchanger (NCX) (Flik and Verbost, 1993; Flik et al., 1995), and energy-consuming maintenance of ion gradients by Na^+^/K^+^-ATPase on the basolateral membrane (Hwang et al., 2011). In contrast, the majority of Ca^2+^ efflux (69–86%) is thought to be mediated by paracellular transport through the gill epithelium (Flik et al., 1986), which is regulated by permeability between cells.

Teleosts are poikilothermic animals, and their body temperatures fluctuate with changes in the environmental temperature. Ionic imbalance in fish cells may occur as a result of decreased environmental temperatures because the ion transporters generally display a higher temperature coefficient (Q_10_ = 2–3) than does permeability (Q_10_ = 1–2) between fish cells (Cossins et al., 1995). Under hypothermic conditions, plasma Ca^2+^ levels were previously reported to be decreased in both carp (*Cyprinus carpio*) (Houston et al., 1970) and in goldfish (*Carassius auratus*) (Houston and Koss, 1982). However, the mechanisms governing the decrease of whole body plasma Ca^2+^ are not clear. Until now, the molecular response of Ca^2+^ regulation to cold challenge has mostly been studied with regard to Ca^2+^-ATPase kinetics in the sarcoplasmic reticulum, for which it was reported that activity is upregulated in white muscle of carp (Ushio and Watabe, 1993; Vornanen et al., 1999) and in heart of trout (Aho and Vornanen, 1998). Other adaptive mechanisms of Ca^2+^ regulation have not been studied in detail.

Environmental factors, such as pH, ion levels and pollutants, have been well-documented to impact whole-body Ca^2+^ regulation, resulting in growth inhibition or even death of fish (Rodgers, 1984). However, only a few studies have addressed the effects of hypothermic environments on whole-body Ca^2+^ regulation in goldfish (Takagi et al., 1989). In this study, we first compared the regulation of whole-body Ca^2+^ fluxes in response to low-temperature challenge of eurythermic goldfish and stenothermic tilapia (*Oreochromis mossambicus*). The tilapia, a tropical species, lives exclusively in warm temperature waters, while the goldfish is a cyprinid that has the ability to survive in a wider temperature range. Based on this key distinction, we proposed that these two species may exhibit different compensatory mechanisms for whole-body Ca^2+^ regulation during acclimatization to a hypothermic environment. Whole-body Ca^2+^ influx, efflux and net flux rates, as well as kinetic constants (*K_m_* and *V_max_*) for Ca^2+^ influx were examined in these two species after acute and chronic exposures to low temperature. In order to assess potential mechanisms by which Ca^2+^ flux was modulated by low temperature, we probed the mRNA expression levels of the major Ca^2+^ transporters, ECaC, NCX, and PMCA, on the gill in both fishes after cold acclimation. Furthermore, we measured the activity, quantity and sensitivity of Na^+^/K^+^-ATPase, one of the major active transporters involved in maintaining epithelial cell ionic gradients, including those that drive Ca^2+^ transport (Hwang et al., 2011).

## Materials and Methods

### Animals

Juvenile tilapia (*O. mossambicus*), weighing 5–10 g, were obtained from a laboratory stock, and goldfish (*C. auratus*), weighing 5–12 g, were obtained from local aquariums. All fish were reared in aerated local tap water [(Ca^2+^) = 0.2 mM] at 27–29°C with a 12L:12D photoperiod. The fish were fed a daily diet of commercial pellets (Fwu-Sow, Taipei, Taiwan). All animal experiments and handling procedures were overseen by the Institutional Animal Care and Use Committee (Protocol No. 104078) at National Cheng Kung University, Taiwan. The 3R policy (Reduce, Replace, and Refine) for animal handling was followed, according to guidance from the Ministry of Science and Technology, Taiwan.

### Calcium and Sodium Content in Tissue and Media

At the end of the 2-week acclimation periods, fish were euthanized with an overdose of anesthetic (MS222, 0.38 mM). Body weights were measured, and then fish were dried and digested with 13.1 N HNO_3_ at 60°C overnight. Digested solutions and environmental water samples were diluted with double-deionized water and were analyzed with an atomic absorption spectrophotometer (Hitachi Z-8000, Tokyo, Japan) to measure calcium and sodium concentrations. Calcium and sodium standard solutions (Merck, Darmstadt, Germany) were used to create standard curves for the measurements.

### Calcium Flux

Ca^2+^ flux rates were determined according to previous methods (Wood, 1992; Chen et al., 2003) with some modifications. Tracer media were prepared by adding ^45^Ca^2+^ (Amersham, Piscataway, NJ, United States) into aerated tap water [(Ca^2+^) = 0.2 mM], such that the specific activity of the media was 510,000–540,000 cpm μmole^-1^. The tracer media (100 ml) in flux chambers was gently aerated without filter equipment, and the water quality (pH and ion concentration) showed no significant change during the period of incubation. Preliminary experiments indicated that the radioisotope adheres nonspecifically to the surfaces of flux chamber and the fish, but this non-specific redistribution of material was saturated within the first 10 min of incubation. In addition, the plot of accumulated radioisotope in fish against time was linear over the first 6 h, and the calculated influxes at each period within the first 6 h were constant. Therefore, water samples (200 μL; two samples for each determination) were collected 10 min and 6 h 10 min after incubation in order to exclude the effects of non-specific adsorption. Counting solution (Fluoran-safe Scintran, BDH, Poole, United Kingdom) was added to water samples from the ^45^Ca^2+^ media, and then radioactivity was counted with a β-counter (LS6500, Beckman, Fullerton, CA, United States). The Ca^2+^ influx (J_in_, nmole g^-1^ h^-1^) was determined by the formula:

$$J_{in}=({\left[R\right]}_{i}\,\;V_{i}-{\left[R\right]}_{f}\,\;V_{f})÷\left\{1/2({\left[R\right]}_{i}/{\left[{Ca}^{2+}\right]}_{i}+{\left[R\right]}_{f}/{\left[{Ca}^{2+}\right]}_{f})tW\right\}$$

where [R]_i_ and [R]_f_ (cpm mL^-1^) refer to initial (10 min) and final (6 h 10 min) concentrations of radioactivity in the tracer media, V_i_ and V_f_ (mL) refer to volumes of the initial and final tracer media, [Ca^2+^]_i_ and [Ca^2+^]_f_ were the initial and final concentrations of Ca^2+^ in the tracer media, *t* (6 h) is incubation time, and *W* is body weight of fish in grams.

Water samples (200 μL; two samples for each determination) were collected 10 min and 6 h 10 min after the beginning of the incubation. The Ca^2+^ net flux (J_net_, nmole g^-1^ h^-1^) was determined by the formula:

$$J_{net}=({\left[{Ca}^{2+}\right]}_{i}\,\;V_{i}-{\left[{Ca}^{2+}\right]}_{f}\,\;V_{f})÷\left(tW\right)$$

Efflux rates (J_eff_, nmole g^-1^ h^-1^) were determined by the equation:

$$J_{eff}=J_{in}-J_{net}\,$$

The flux experiments were repeated three times.

### Ca^2+^ Influx Kinetics

The protocol for measuring Ca^2+^ influx kinetics was according to earlier experiments (Hwang et al., 1996; Chang et al., 1998) with some modifications. Tilapia and goldfish were individually introduced into flux chambers after being rinsed in artificial Ca^2+^-free solution (Na^+^ 0.5 mM, Mg^2+^ 0.16 mM, K^+^ 0.3 mM) at room temperature in order to remove extra Ca^2+^ ions from the body surface. Flux chambers were filled with tracer media (20 ml) that was prepared by adding CaSO_4_ to artificial Ca^2+^-free solution with final concentrations of Ca^2+^ ranging from 0.01 to 5 mM. ^45^Ca^2+^-labeled CaCl_2_ stock solutions were also added to the prepared Ca^2+^ solutions so that the same Ca^2+^/^45^Ca^2+^ ratio was maintained in each solution. Flux experiments and calculation of Ca^2+^ influxes at different external Ca^2+^ levels were performed for 2 h. Eadie–Hofstee plots were used to calculate the values of *K_m_* (Michaelis constant) and *V_max_* (maximum velocity) (**Supplementary Figure S1**). These values were applied to the Michaelis–Menten equation:

$$J_{in}=(V_{\max}\left[{Ca}^{2+}\right])÷\left([{Ca}^{2+}]+K_{m}\right)$$

where [Ca^2+^] is the concentration of Ca^2+^ in the tracer media. The influx kinetics experiments were repeated three times.

### Na^+^/K^+^-ATPase Activity, Protein Expression and Sensitivity

Gill Na^+^/K^+^-ATPase activity was measured as previously described (Hwang et al., 1989; Lee et al., 2003). Fish gill tissue (50 mg) was homogenized in 500 μL extraction buffer (33 mM Tris-HCl, 0.25 M sucrose, 25 mM EDTA, 9.6 mM sodium deoxycholate) on ice and were centrifuged at 1000 × *g* for 20 min at 4°C. Ten microliters supernatant was mixed with reaction buffer (0.12 M NaCl, 74 mM KCl, 15.7 mM MgCl_2_, 98 mM imidazole, 5 mM ATP, pH 7.6) with or without 0.5 mM ouabain for 30 min at 37°C. The reactions were stopped by 8.5% trichloroacetic and were centrifuged 4000 × *g* for 10 min at 4°C. Supernatant (500 μl) was mixed with 1000 μl colorization buffer (0.83% acid molydate, 2.7% SDS, 0.0017% ANSA) for 30 min at 20°C. The concentration of inorganic phosphate was determined by spectrophotometry (Hitachi). Protein concentration was determined by the Bradford assay, and BSA was used as a standard. An inorganic phosphate standard curve was prepared with Na_2_HPO_4_ in concentrations ranging from 0 to 80 μg/ml. The Na^+^/K^+^-ATPase activity was calculated as the concentration differences of inorganic phosphate inhibited by ouabain.

The Na^+^/K^+^-ATPase (1:2000, anti-α5 subunit, John Hopkins University) or β-actin (1:2000, Abcam, ab8227) primary antibodies and HRP-conjugated goat anti-mouse (1:5000, Jackson ImmunoResearch) or HRP-conjugated goat anti-rabbit (1:3000, GeneTex) secondary antibodies were used for western blotting analysis as previously described (Lee et al., 2003). Immunoblots were quantified using Image J. Na^+^/K^+^-ATPase sensitivity was measured according to an earlier report (Schwarzbaum et al., 1992), where it was calculated from the half maximal inhibitory concentration of Ouabain (IC_50_) against Na^+^/K^+^-ATPase activity.

### Quantitative RT-PCR

Quantitative (real-time) RT-PCR was performed and analyzed as described in a previous study (Wang et al., 2017). Gill cDNA were synthesized by the Transcriptor First Strand cDNA Synthesis Kit (Roche) using random hexamer and poly(dT) primers. Real-time PCR was performed with 2X SYBR green master mix (Promega) and a StepOne Real-Time PCR System (Applied Biosystems, Thermo Fisher Scientific). The primers used for *NCX1b* (*Na^+^, Ca^2+^ exchanger 1b*), *ECaC* (*epithelial Ca^2+^ channel*), *PMCA* (*plasma membrane Ca^2+^ ATPase*), and internal controls of *ribosomal protein L7* (*RPL7*) for tilapia or *18S ribosomal RNA* (*18S rRNA*) for goldfish are shown in **Supplementary Table S1**. Experiments were repeated three times, and groups were compared by Student’s *t*-test.

### *In situ* Hybridization and Immunostaining

*In situ* hybridization was performed following a previous study (Lin et al., 2016). Briefly, gills were fixed overnight with 4% paraformaldehyde in PBS. The fixed gills were washed with PBS and cryoprotected in 30% sucrose prior to embedding in OCT (Tissue-Tek). The gills were then sectioned longitudinally at 15 μm. Before *in situ* hybridization, the slide-mounted gill sections were air-dried and rehydrated by series of methanol and PBST (0.1% Tween) mixtures. The rehydrated slides were incubated in the pre-hybridization buffer for 2 h and hybridized with RNA probes at 70°C overnight. The hybridized slides were washed with mixture of hybridization buffer and 20X SSC, followed by 0.2X SSC at 70°C. Next, the gill slides were washed with mixture of 0.2X SSC and PBST at room temperature. The washed slides were transferred into blocking buffer containing 2% normal goat serum and 2 mg/ml BSA for 2 h at room temperature. The samples were incubated with alkaline phosphatase-conjugated anti-DIG antibody (1:5000, Roche) overnight at 4°C. The tissues were washed with PBST six times for 15 min each. The washed samples were then transferred into alkaline Tris buffer, containing 0.2 M Tris HCl, pH 9.5, 0.1 M MgCl_2_, 0.2 M NaCl and 0.2% Tween 20. Tissues were labeled with a mixture of 225 μg/ml NBT (Nitro Blue Tetrazolium) and 175 μg/ml BCIP (5-Bromo 4-Chloro 3-indolyl Phosphate). The labeling reaction was stopped with stop solution, containing 1 mM EDTA, 0.1% Tween 20 in 1X PBS, pH 5.5. After *in situ* hybridization, the gill slides were washed with PBST three times for 15 min each; then slides were incubated with 3% BSA in PBST for 2 h at room temperature. Slides were transferred into Na^+^/K^+^-ATPase (1:400, anti-α5 subunit, John Hopkins University) antibody and incubated overnight at 4°C. The gill sections were washed with PBST six times for 10 min each, and then incubated with Alexa 488-conjugated goat anti-mouse antibody (Thermo Fisher Scientific, 1:400) for 2 h at room temperature. The stained gill slides were mounted with VECTASHIELD antifade mounting medium. Images were captured with a Zeiss LSM 780 confocal microscope and the colocalization of NKA and PMCA signaling was determined using the profile function in Zen2010 software (Zeiss). The Na^+^/K^+^-ATPase, PMCA or double-stained cell numbers per sections were counted and recorded. The cell number was recorded from one middle section per gill filament, three gill filaments per fish and three fish from each experimental group. Values are expressed as mean ± S.D. Differences were considered significant for *p* ≤ 0.05, *n* = 3, as calculated by Student’s *t*-test. Fluorescence intensities of Na^+^/K^+^-ATPase immunostaining and DAPI were measured using the histology function in Zen2010 software (Zeiss). The Alexa 488 intensity was normalized to DAPI intensity in the same area of the gill section.

### Statistics

All values are shown as mean ± S.D. Data were first analyzed with Shapiro–Wilk’s test for normality and Levene’s test for equality of variance before further analysis. One-way ANOVA with *post hoc* Tukey’s multiple comparison was used to compare the Ca^2+^ influx, net flux and efflux kinetics among groups in the same species. Na^+^/K^+^-ATPase expression, activity, Ca^2+^ transporter expression and cell numbers were compared by Student’s *t*-test. *P* < 0.05 was considered statistically significant.

### Acclimation Experiments

#### Acute Exposure to Low Temperature

Both tilapia and goldfish were acclimated in tap water at 25 ± 1°C for 2 weeks. After acclimation, fish (*n* = 8 per group) were directly transferred to 25°C and 15°C tracer media, after which the Ca^2+^ flux rates were measured.

#### Long-Term Acclimation to Low Temperature

Tilapia and goldfish were randomly divided into three groups per species and the temperature was gradually decreased by 2°C per day from 25°C to 15°C, after which the fish were acclimated for 2 weeks with the filter equipment. During the acclimation period, the fish from both temperature groups were not fed. During the cold acclimation, tilapia exhibited decreased motility with 10–20% mortality, but goldfish did not. At the end of acclimation, the first group of each species (*n* = 8) was used to measure Ca^2+^ influx, efflux and net flux at the acclimation temperature. The second group of fish (*n* = 7 for tilapia and *n* = 9 for goldfish) was digested and used for the whole-body Ca^2+^ concentration determinations. The third group of acclimated fish (*n* = 4) was used to determine the Ca^2+^ influx kinetics at 25°C directly.

#### Minimization of Handling Effects on Ca^2+^ Flux Determination

In order to minimize handling effects on the Ca^2+^ flux determinations, fish were handled according to following procedure. After being maintained in the acclimation tank, individual fish were placed in the opaque flux chambers (100 ml) with recirculated dechlorinated local tap water for 3 days in order to exclude the handling effects (Dharmamba and Maetz, 1972). At the beginning of the determination period, flow to the chamber was stopped, ^45^Ca as CaCl_2_ was added, and water samples were collected for simultaneous determination of Ca^2+^ influx and efflux. In the long-term acclimation experiments, Ca^2+^ fluxes were determined in the chamber at the same temperature as the acclimation. During the acute low-temperature acclimation, the water temperature in the chamber was decreased from 25°C to 15°C over the course of 1 h and then the Ca^2+^ flux determination procedure was initiated.

## Results

### Whole-Body Calcium and Sodium Content

After long-term cold acclimation, whole-body calcium and sodium contents were reduced significantly both in goldfish and tilapia (**Table 1**). However, the calcium reduction was less severe in goldfish (8.7% reduction compared with the 25°C control) than in tilapia (17.2%). The same was true for the reduction in sodium content (11.6% in goldfish and 25.2% in tilapia). This result indicated that goldfish can more effectively maintain whole-body calcium and sodium contents in cold environments.

**Table 1:** Whole-body calcium and sodium content in tilapia and goldfish after 2-week cold acclimation.

	Tilapia		Goldfish	
Acclimation temperature (°C)	25	15	25	15
Whole-body calcium content (mmole/g)	0.35 ± 0.02	0.29 ± 0.03^∗^ (82.8%)	0.23 ± 0.03	0.21 ± 0.03^∗^ (91.3%)
Whole-body sodium content (μmole/g)	50.0 ± 4.8	37.4 ± 4.8^∗^ (74.8%)	42.7 ± 6.9	37.7 ± 2.2^∗^ (88.4%)


*^∗^Significant decrease compared to the 25°C acclimated group in the same species (Student’s *t*-test, *p* < 0.05). The percentage values are the relative calcium content comparing the 15–25°C acclimated groups in same species. Mean values ± S.D. were derived from tilapia (*n* = 7) and goldfish (*n* = 9).*

### Ca^2+^ Flux Rates After Acute and Chronic Cold Acclimation

**FIGURE 1:**
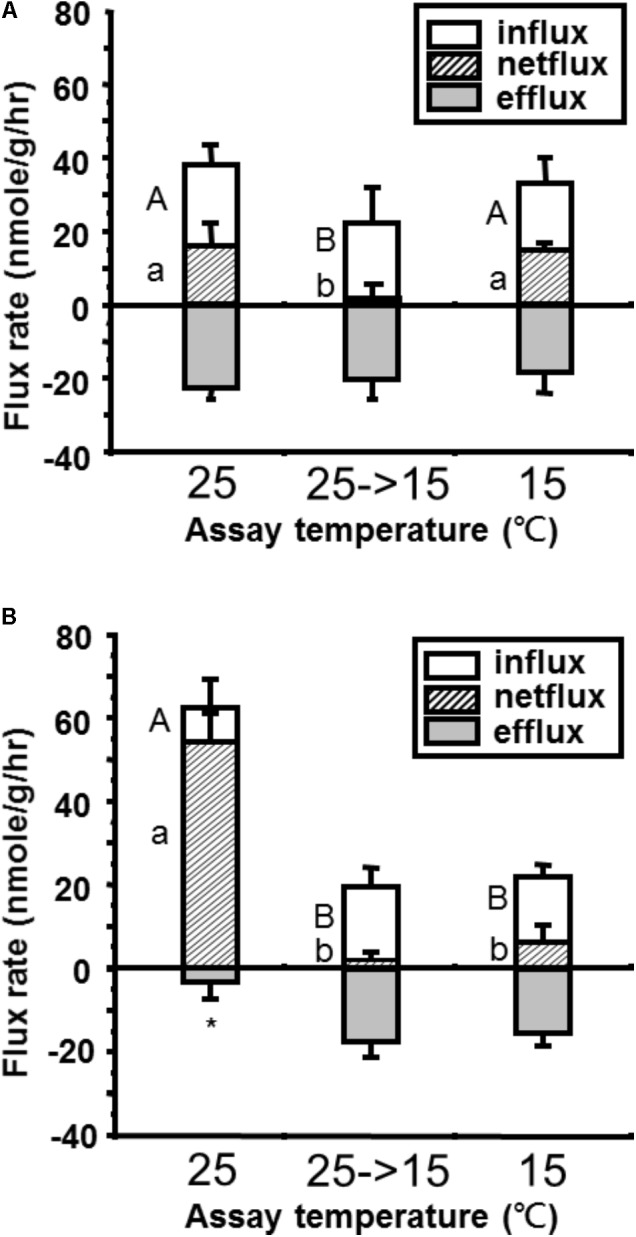
Comparison of whole-body Ca^2+^ flux rates in **(A)** goldfish and **(B)** tilapia after short term (25°C→15°C) or long-term (15°C) cold acclimation. One-way ANOVA, Tukey’s multiple-comparison. ^AB^*p* < 0.05 compared to short- and long-term acclimation Ca^2+^ influx, ^ab^*p* < 0.05 compared to short- and long-term Ca^2+^ net flux and ^∗^*p* < 0.05 compared to short- and long-term Ca^2+^ efflux (*n* = 8) are significant different.

Upon acute exposure to low temperature (15°C), Ca^2+^ influx was reduced significantly in both tilapia and goldfish (**Figure 1**). However, the reduction in tilapia (from 62 to 20 nmole g^-1^ h^-1^ = 68% reduction, **Figure 1B**) was more extreme than in goldfish (from 39 to 23 nmole g^-1^ h^-1^ = 41% reduction, **Figure 1A**). This result indicated that the Q_10_ between 25°C and 15°C for Ca^2+^ influx was higher in tilapia than goldfish. After long-term acclimation, goldfish showed a complete recovery of Ca^2+^ influx. However, no significant compensation for reduced Ca^2+^ influx was detected in tilapia after long-term cold acclimation.

During acute and long-term cold exposure, goldfish did not show any significant change in Ca^2+^ efflux rates compared with the 25°C control group (**Figure 1A**). In contrast, significant increases in Ca^2+^ efflux were detected in tilapia after both acute and long-term cold exposure (**Figure 1B**).

Similar to Ca^2+^ influx, goldfish maintained a positive but very low level of Ca^2+^ net flux upon acute exposure and a complete recovery after long-term acclimation (**Figure 1A**). However, the decrease of Ca^2+^ net flux upon acute cold exposure in tilapia did not significantly recover after long-term exposure (**Figure 1B**).

### Ca^2+^ Influx Kinetic Constants After Cold Acclimation

**FIGURE 2:**
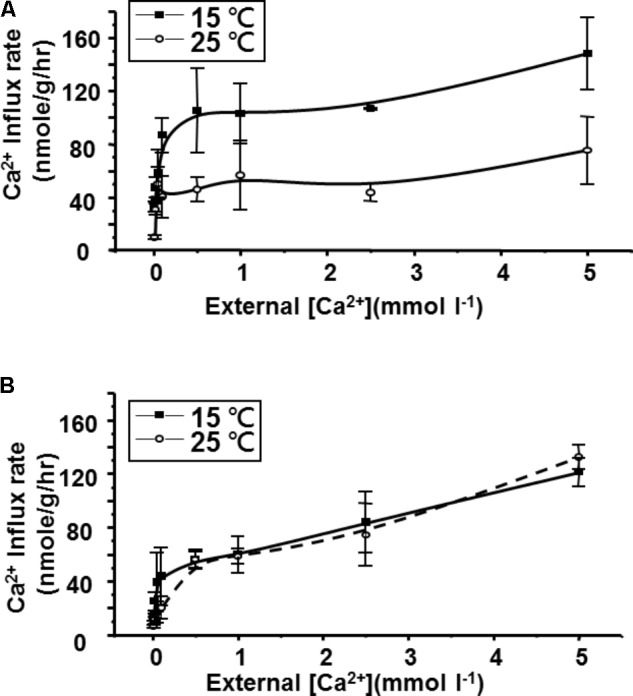
Comparison of Ca^2+^ influx kinetics after 2 weeks cold (15°C) acclimation or control (25°C) for **(A)** goldfish and **(B)** tilapia. Calcium influx was measured in tracer media over a range of calcium concentrations. The mean (closed squares for 15°C acclimated and open circles for 25°C acclimated) and deviation (bar) were obtained from four fish for each point (*n* = 4).

Ca^2+^ influx rates, determined at different Ca^2+^ concentrations, displayed typical Michaelis–Menten kinetics in both goldfish (**Figure 2A**) and tilapia (**Figure 2B**) after acclimation at two different temperatures. As shown in **Table 2**, 2 weeks of hypothermic acclimation resulted in a decreased *K_m_* (Michaelis constant) and an increased *V_max_* (maximum velocity) for Ca^2+^ influx in goldfish. However, neither the *K_m_* nor the *V_max_* were changed after the same treatment in tilapia. These results indicated that binding affinity and maximum velocity for Ca^2+^ transport are both increased after cold acclimation in goldfish but not in tilapia.

**Table 2:** Kinetic constants, *K_m_* and *V_max_* for the calcium influx (J_in_) in tilapia and goldfish after cold acclimation.

Species	Acclimation temperature (°C)	*K_m_* (mM)	*V_max_* (nmole g^-1^ h^-1^)
Tilapia	25	0.07 ± 0.02	52.4 ± 15.4
Tilapia	15	0.04 ± 0.02	67.9 ± 22.3
Goldfish	25	0.05 ± 0.01	66.4 ± 3.0
Goldfish	15	0.02 ± 0.01^∗^	105.4 ± 20.0^∗^


*Values shown are mean ± S.D. Calcium influx rates were measured at 25°C. ^∗^Significant difference between 15 and 25°C acclimated groups in the same species (Student’s *t*-test, *p* ≤ 0.05, *N* = 4).*

### Na^+^/K^+^-ATPase Activity, Protein Expression and Sensitivity

**FIGURE 3:**
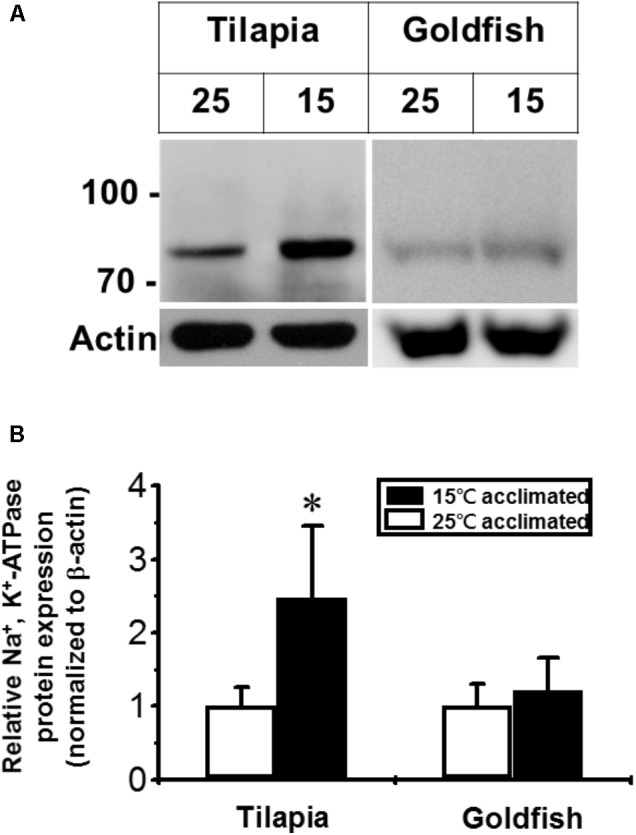
Effect of cold acclimation on the expression of Na^+^/K^+^-ATPase in tilapia or goldfish gill. **(A)** The abundance of Na^+^/K^+^-ATPase was determined by western blotting analysis. Different numbers (25 and 15) indicated acclimation temperature (°C). The immunoblot for actin served as an internal control. **(B)** The quantification of the Na^+^/K^+^-ATPase expression. The Na^+^/K^+^-ATPase expression was normalized to β-actin and was also relative to 25°C acclimated group (set as 1). Values shown are mean ± S.D. ^∗^Significant difference between 15°C and 25°C acclimated groups in the same species (Student’s *t*-test, *p* ≤ 0.05, *n* = 4).

**FIGURE 4:**
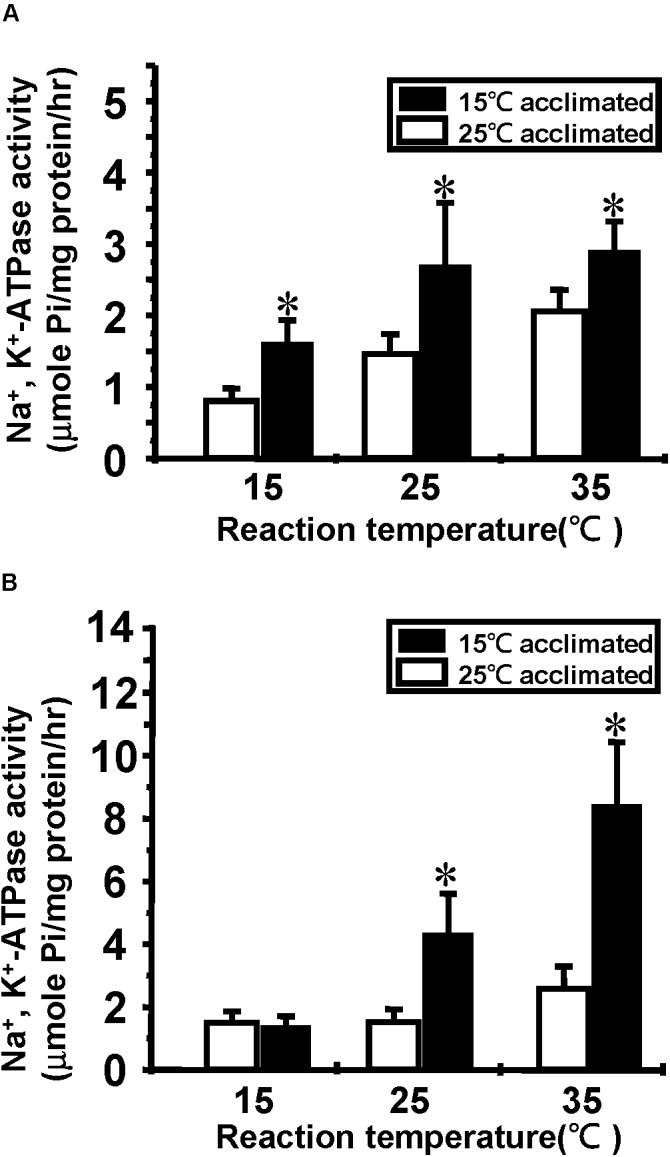
Effect of cold acclimation on the Na^+^/K^+^-ATPase activity in tilapia or goldfish gill. Na^+^/K^+^-ATPase activity was determined in 15°C and 25°C acclimated **(A)** goldfish or **(B)** tilapia at three reaction temperatures (15, 25, and 35°C). Asterisks indicate significant differences between the two acclimation temperature groups with Student’s *t*-test, *p* < 0.05, *n* = 4.

Na^+^/K^+^-ATPase is one of the most important molecules in the control of active ion absorption in freshwater fish (Hwang et al., 2011). Changes in Na^+^/K^+^-ATPase properties, including ouabain sensitive activity, density and turnover rate, have been suggested to play important roles in teleost cold acclimation (Gibbs, 1995). Therefore, we determined the Na^+^/K^+^-ATPase activity and protein abundance in goldfish and tilapia after cold acclimation. The results showed that after long-term cold acclimation, sodium pump content was increased in the gill tissue of tilapia but not goldfish (**Figure 3**). However, Na^+^/K^+^-ATPase activity was enhanced in cold acclimated goldfish (**Figure 4**). Interestingly, both cold acclimated tilapia (**Figure 4B**) and goldfish (**Figure 4A**) showed increased activity when measurements were made at 25°C and 35°C. However, only cold acclimated goldfish showed increased activity with a 15°C reaction temperature. In order to test whether Na^+^/K^+^-ATPase has similar ion binding affinity after cold acclimation, Na^+^/K^+^-ATPase sensitivity, as determined by IC_50_ of ouabain was measured (**Table 3**). The goldfish showed a significant decrease in ouabain IC_50_ after cold acclimation.

**Table 3:** Inhibitory concentrations of Ouabain (IC_50_) for Na^+^/K^+^-ATPase activity in tilapia and goldfish gill after cold acclimation.

Species	Acclimation temperature (°C)	Ouabain concentration (mM)
Tilapia	25	1.3×10^-7^
Tilapia	15	1.3×10^-7^
Goldfish	25	4.6×10^-7^
Goldfish	15	2.0×10^-7^^∗^


*Values shown are mean ± S.D. ^∗^Significant difference between 15 and 25°C acclimated group in the same species (Student’s *t*-test, *p* < 0.05, *N* = 4).*

### Transcript Expression Levels of Calcium Related Transporters

**FIGURE 5:**
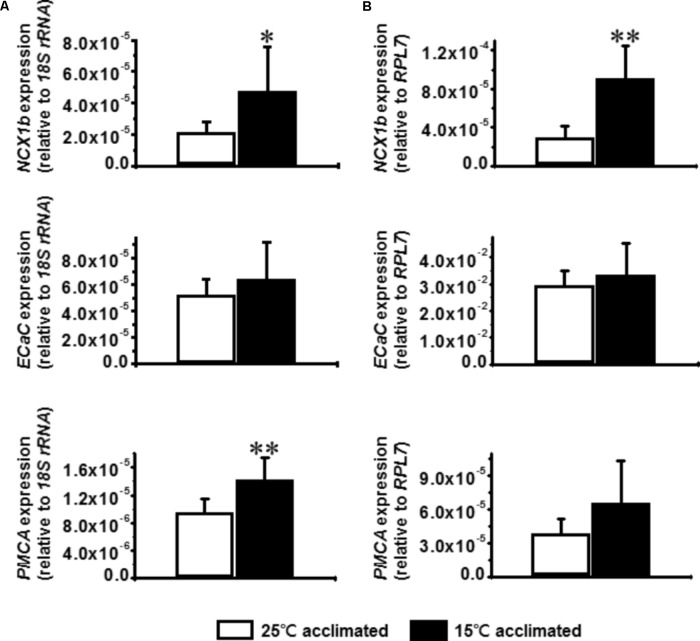
Effect of cold acclimation on the expression of calcium transporters in tilapia or goldfish gill. Relative mRNA expression of *Na^+^/Ca^2+^ exchanger 1b* (*NCX1b*), *epithelial Ca^2+^ channel* (*ECaC*), *plasma membrane Ca^2+^ ATPase* (*PMCA*) in **(A)** goldfish or **(B)** tilapia gill were measured by real-time quantitative PCR. The expression of *18S ribosomal RNA* (*18S rRNA*) was used as an internal control for goldfish and *ribosomal protein L7* (*RPL7*) was used for tilapia. Student’s *t*-test, ^∗^*p* ≤ 0.05, ^∗∗^*p* ≤ 0.01 compared to 15°C acclimated fish. *n* = 6 per group.

The calcium related transporters, NCX1b, ECaC, and PMCA are located on the gill and are thought to constitute the major driving force for calcium absorption in freshwater fish (Hwang et al., 2011). Increased expression of ECaC in zebrafish gill after cold acclimation has been previously reported (Chou et al., 2008), suggesting that increased expression of calcium transporters can be utilized as a cold acclimation strategy. Therefore, the mRNA expression levels of *NCX1b*, *ECaC*, and *PMCA* were determined after cold acclimation in tilapia and goldfish. The results showed that the expression levels of *NCX1b* but not *ECaC* are increased both in tilapia and goldfish after cold acclimation (**Figures 5A,B**). Interestingly, the expression of *PMCA* was increased significantly only in goldfish (**Figure 5A**), suggesting that increase of *PMCA* mRNA may be an especially important factor for successful cold acclimation.

### The Effect of Cold Acclimation on Ionocyte Numbers

**FIGURE 6:**
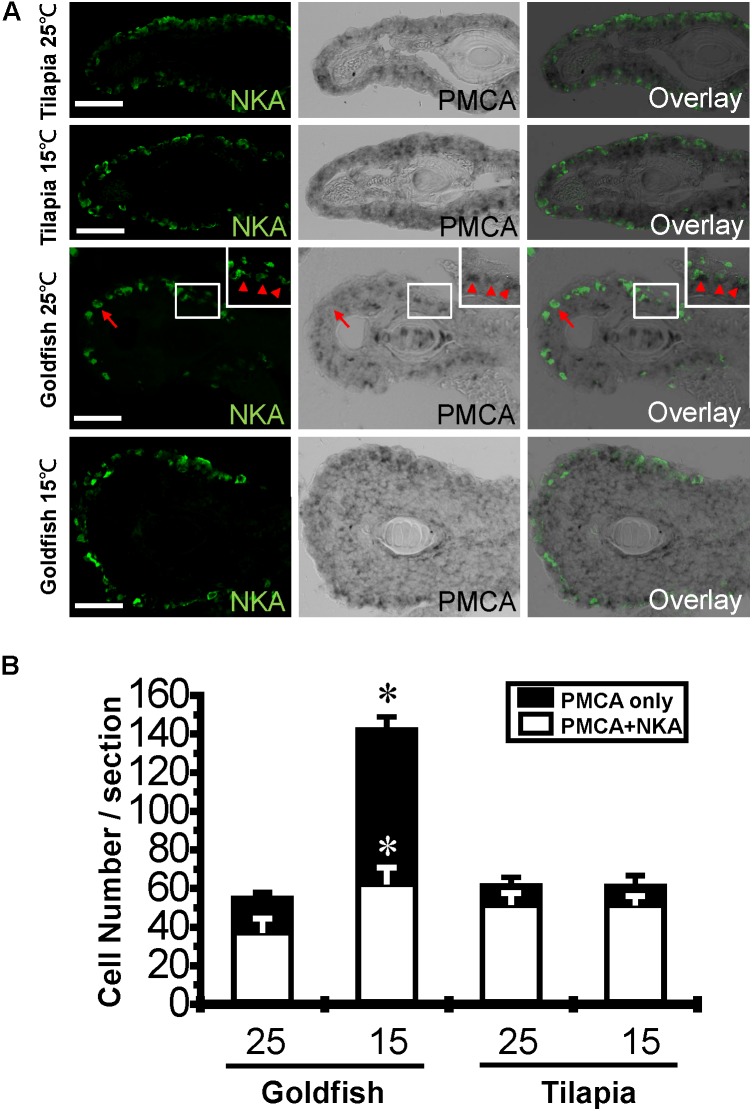
Effect of cold acclimation on the Na^+^/K^+^-ATPase and PMCA-expressing ionocytes. Frozen sections of 25°C (**A**, first row) and 15°C (**A**, second row) acclimated tilapia, and 25°C (**A**, third row) and 15°C (**A**, fourth row) acclimated goldfish gills were stained with Na^+^/K^+^-ATPase (NKA) antibody and hybridized with PMCA RNA probes. Inset shows an enlarged picture of three co-stained cells. The numbers of PMCA only cells (arrow) or the double-stained (NKA/PMCA) cells (arrowhead) were averaged from three fish for each treatment (*n* = 3) **(B)**. The cell numbers of three sections from different gill filaments of each fish are expressed as mean ± S.D. The means of control temperature (25°C) and cold-acclimated (15°C) groups from the same species were compared by Students’ *t*-test, ^∗^*p* ≤ 0.05. Scale bar, 20 μm.

Surface area and cell numbers of gill ionocytes were previously reported to be increased after cold acclimation in goldfish (Mitrovic and Perry, 2009). Therefore, in addition to detecting the abundance of gill transporter proteins by western blot, we detected the effects of cold acclimation on ionocyte numbers and the fluorescence intensity per cell by Na^+^/K^+^-ATPase immunostaining. The fluorescence intensity was increased in tilapia after cold acclimation (**Supplementary Figure S2**), suggesting that the increase of Na^+^/K^+^-ATPase abundance detected by western blotting in tilapia may due to increased protein density per cell instead of cell number. In addition, the transcription of PMCA, which is the major Ca^2+^ active transporter, was increased significantly in goldfish but not in tilapia after cold acclimation. Previous studies have shown that PMCA and ECaC-expressing cells are not completely colocalized in zebrafish embryos (Liao et al., 2007). Therefore, we detected PMCA expression by *in situ* hybridization. The result indicated that both the number of Na^+^/K^+^-ATPase and PMCA co-expressing cells and the number of PMCA only cells were upregulated in goldfish (**Figures 6A,B** and **Supplementary Figure S3**) but not in tilapia after cold acclimation.

## Discussion

This study is the first to compare the effects of hypothermic exposure on the whole-body Ca^2+^ balance in eurythermic and stenothermic teleosts. The major findings are as follows. (1) Upon acute cold exposure, stenothermic tilapia showed a higher Q_10_ (3.1) for whole-body Ca^2+^ influx than goldfish (1.7). (2) After long-term cold acclimation, goldfish recovered Ca^2+^ net flux due to compensatory increases in influx. However, recovery of influx was not detected in tilapia. (3) After long-term cold acclimation, goldfish showed compensatory increases in *V_max_* and decreases in *K_m_* for Ca^2+^ influx, while no compensation was observed after cold acclimation in tilapia. (4) This compensatory response in goldfish may be related to the observed upregulation of Na^+^/K^+^-ATPase activity and PMCA expression, as well as the increased number of PMCA-expressing cells.

Upon acute hypothermic exposure, the larger Q_10_ of Ca^2+^ influx and lack of recovery for net uptake in tilapia indicated that the Ca^2+^ active transport mechanism is more sensitive to low environmental temperature in tilapia than in goldfish. Acute hypothermic effects on enzyme activities have been well-studied in various cells or tissues. Homeotherms and poikilotherms were compared on the basis of their mitochondrial succinate oxidase activities in rat and trout livers (Lyons and Raison, 1970), or in another study, the NCX in sarcolemma from bullfrog, dog, and rabbit were compared (Bersohn et al., 1991). Stenotherms and eurytherms have also been compared, including a study on sliver carp (*Hypophthalmichtys molitrix*) and subtropical tilapia (*Tristramella simonis simonis*) intestinal alkaline phosphatase (Gelman et al., 1992). Based on Arrhenius plots for enzyme activity, temperature dependence of enzymes from hibernating mammals are distinguishable from non-hibernating animals and eurythermic fishes are different to stenothermic fishes (Raison and Lyons, 1971; Gelman et al., 1992). In addition, by comparing the whole body Na^+^ uptake, Gonzalez and Mcdonald (2000) also demonstrated that stenothermic rainbow trout (*Oncorhynchus mykiss*) was more sensitive than eurythermic shiner (*Notropis cornutus*) upon acute cold exposure. Therefore, our current data agree with the previous literature, suggesting that animals living within a narrower range of temperatures are generally more sensitive to temperature fluctuations.

The gill has been shown to be the major organ responsible for whole-body Ca^2+^ influx from the environment, and this process relies on energy-consuming transcellular transport mechanisms (Perry, 1997; Hwang et al., 2011). In the present study, a compensatory mechanism for whole-body Ca^2+^ balance (enhanced Ca^2+^ influx that was characterized by increased *V_max_* and decreased *K_m_*) was detected in goldfish after long-term cold acclimation. Several mechanisms have been proposed to compensate for reduced enzyme activity during cold acclimation. These include upregulation of enzyme density, post-translational modifications or induced synthesis of kinetically distinct protein variants (Cossins et al., 2007). We demonstrated that the expression of PMCA is upregulated in goldfish after cold acclimation and may reflect an increase of cell number (PMCA-expressing cells with or without NKA staining). Wright et al. (2016) have reported that common carp, a cyprinid fish, has two types of NKA-rich ionocytes, one is α5-positive and the other is negative. Therefore, using our methodology, the NKA and PMCA double-positive cell numbers may have been underestimated in cold acclimated goldfish (**Figure 6B**). However, this observation should only account for the increase of *V_max_* of Ca^2+^ influx but not the affinity. Although there is still no direct evidence showing that different Ca^2+^ transporter isoforms participate in cold acclimation, a few lines of evidence have suggested it may be an efficient mechanism (Hazel and Prosser, 1974). First, differential isoform expression for proteins related to muscle contraction – including myosin heavy chain – have been detected during thermal acclimation in carp, likely helping the fish cope with environmental challenges (Gerlach et al., 1990; Watabe, 2002). Second, different isozymes of Ca^2+^-transport proteins have been identified, and these isozymes are characterized by a *U*-shaped *K_m_*-temperature curve (Hazel and Prosser, 1974; Withers, 1992) and variable minimal *K_m_*. Thus, the decreased *K_m_* that we observed in goldfish may potentially be explained by altered expression of different isozymes in Ca^2+^-transport. In the present study, we identified upregulation of *PMCA* expression possibly by increasing cell number in goldfish as a likely important factor in the regulation of Ca^2+^ influx kinetics. The primer pairs used for real-time detection and *in situ* hybridization of *PMCA* are specific to tilapia (*O. mossambicus*) and goldfish, respectively. However, the isoforms of *PMCA* in other *Teleostei* species are known to be highly homologous (more than 70% identity of nucleotide sequences). Since tilapia (*O. mossambicus*) and goldfish are not commonly used model species, the sequences of *PMCA* isoforms are not available. Therefore, our data cannot exclude the possibility that upregulation of one or several *PMCA* variants may be used as a strategy for goldfish acclimation. Furthermore, identification and enzymatic characterization of the specific isoform of *PMCA* that is regulated in response to cold exposure in goldfish still needs further exploration.

In addition to altering the transporters, the regulation of protein microenvironments has also been proposed as a strategy for cold acclimation. The term “homeoviscous adaptation” refers to modifications in the fatty acid composition of phospholipids (or the degree of unsaturation) in order to maintain membrane fluidity (or viscosity) (Cossins et al., 1995), and this adaptation has been shown to be an important factor for regulating the activity of membrane enzymes. Changes in phospholipid (Hazel and Carpenter, 1985) or cholesterol content (Hazel and Carpenter, 1985; Robertson and Hazel, 1995) have been shown in rainbow trout gills or carp brain upon cold acclimation. Although a previous study showed a lack of homeoviscous adaptations in the sarcoplasmic reticulum of cold acclimated goldfish muscle (Cossins et al., 1978), the results from other studies have suggested decreased activation energy for Mg^2+^/Ca^2+^ ATPase (Johnston, 1979) and increased Ca^2+^ transportation rate (Penny and Goldspink, 1980) can be detected in the same organs of the same species and may contribute to maintained locomotor activity (Johnston et al., 1990). The homeoviscous adaptation may, therefore, still provide a possible explanation for Ca^2+^ influx compensation after cold acclimation in the present study.

Based on our whole-body measurements, more Ca^2+^ efflux was observed from tilapia than from goldfish upon acute and long-term cold exposure. Flik et al. (1986) reported that the gill is not only the major location for Ca^2+^ influx, but it is also the major site of efflux in freshwater tilapia. However, the mechanism for Ca^2+^ excretion from the branchial is still not fully described (Hwang et al., 2011). Paracellular efflux from the gill, measured by polyethylene glycol and ammonia, was reported to be decreased in goldfish after cold acclimation, however, Cl^-^ efflux was not changed (Mitrovic and Perry, 2009; Smith et al., 2012). Besides passive diffusion through the gill epithelium, Mackay (1974) has found that urinary ion loss is reduced by acclimation to lower temperatures in goldfish. Thus, it is an interesting and important question to ask if goldfish and tilapia differentially regulate Ca^2+^ permeability in gills and kidneys under hypothermic conditions and whether these effects contribute to changing whole-body Ca^2+^ efflux. Notably, whole-body Ca^2+^ efflux in goldfish was temperature-independent, regardless of acute exposure or long-term acclimation. This is in contrast with what has been reported in fish cells, where passive ion efflux was decreased by reduced environmental temperature. These inconsistent results may be ascribed to the differences between *in vivo* and *in vitro* experiments (Cossins et al., 1995), and imply that acclimation mechanisms for ion balance of whole organisms are not well-modeled by isolated cells.

Whole-body Ca^2+^ and Na^+^ of tilapia and goldfish were decreased after acclimation to low temperature in the present study. Similar decreases in serum Ca^2+^ and Na^+^ were also reported in previous studies of tilapia, carp, and goldfish after cold acclimation (Houston et al., 1970; Allanson et al., 1971; Umminger, 1971; Houston and Koss, 1982; Burton, 1986). Hazel and Prosser (1974) suggested that since energy production will be largely reduced in low temperatures, the lower ion concentrations can reduce the energy expenditure required for the maintaining ion gradients between the body and environment. Therefore, the decrease of whole-body Ca^2+^ and Na^+^ of tilapia and goldfish may be another adaptive strategy for cold acclimation.

Upon cold temperature exposure, upregulation of Na^+^/K^+^-ATPase expression has been detected on the gill in carp (Metz et al., 2003) and goldfish (Mitrovic and Perry, 2009), indicating a compensation strategy for temperature-dependent activity reductions. According to our data, tilapia exhibited increased ATPase activity that may correspond to increased protein quantity, as reflected by increased intensity per cell (**Supplementary Figure S2**), but not changes in sensitivity. These observations suggested that tilapia acclimate to the cold temperature by simply increasing expression of the same protein isoform used at warm temperature (ouabain sensitivity result, **Table 3**). However, this isoform may not perform well in cold temperatures, which could explain why increased activity cannot be detected at a cold reaction temperatures (**Figure 4B**). In contrast to tilapia, Na^+^/K^+^-ATPase quantity in goldfish does not change when detected by western blotting, but shows an increase in cell numbers and activity after cold acclimation (**Figures 3**, **4**, **6**). The inability to detect differences by western blotting may be due tissue dilution effects. If the total cell numbers are altered after cold acclimation (gill remodeling), the overall tissue increase in Na^+^/K^+^-ATPase abundance may not be sufficient to observe by immunoblotting. It was previously suggested by Flik et al. (Metz et al., 2003) that carp (such as goldfish) may change the enzymatic properties of Na^+^/K^+^-ATPase by synthesizing new cold-specific isoforms. If so, the cold-acclimated Na^+^/K^+^-ATPase would show higher activity at cold reaction temperatures, similar to what we observed (**Figure 4A**).

The increase of PMCA in goldfish was tightly associated with upregulation of Ca^2+^ influx *V_max_*. Together, our data and previous studies suggest that effective cold adaptation may require changes in basic protein folding and activities, instead of simple increases of protein amounts.

## Data Availability Statement

All data generated or analyzed during this study are included in this published article and its **Supplementary Information Files**.

## Author Contributions

F-IL and P-PH designed the study. T-YH, C-YWu, H-CT, Y-PC, W-FC, T-CL, C-YWa, J-RL, and F-IL performed the study and analyzed the results. F-IL and P-PH drafted the manuscript. All authors reviewed and approved the final manuscript.

## Conflict of Interest Statement

The authors declare that the research was conducted in the absence of any commercial or financial relationships that could be construed as a potential conflict of interest.

## Abbreviations

ECaC: epithelial Ca^2+^ channel
NCX1b: Na^+^/Ca^2+^ exchanger 1b
PMCA: plasma membrane Ca^2+^ATPase
