# Supplementary material for: Comparison of Calcium Balancing Strategies During Hypothermic Acclimation of Tilapia (Oreochromis mossambicus) and Goldfish (Carassius auratus)
Source: Front Physiol. 2018 Sep 3;9:1224. doi: 10.3389/fphys.2018.01224 (PMC6129941; doi:10.3389/fphys.2018.01224)
Supplement: Supplementary file 1 [file Data_Sheet_1.pdf]

Supplementary Material

Comparison of Calcium Balancing Strategies during Hypothermic Acclimation of Tilapia (*Oreochromis mossambicus*) and Goldfish (*Carassius auratus*)

Tsung-Yu Han[1†], Chien-Yu Wu[2†], Han-Chuan Tsai[1], Yi-Pei Cheng[1], Wei-Fan Chen[1], Tzu-Chien Lin[3], Chia-Yih Wang[3], Jay-Ron Lee[4], Pung-Pung Hwang[4], Fu-I Lu[1, 5*]

1 Supplementary Table

Table S1. Oligonucleotide sequences used for real-time PCR.

Species	Gene	Accession number	Sequences (5′ to 3′)	Product Size (base pairs)
Tilapia	*NCX1b* (Forward)	AY283779	GTGTTGCTTCAGGAAGTCGG	100 bp
	NCX1b (Reverse)		TGCTGATGATGTTGGAGGGG	
	ECaC (Forward)	KU512922	AAAGGAAACGGAGGGAGAGGAGAA	125 bp
	ECaC (Reverse)		CGTCCTGCTCCATCTCCAAACCTAAA	
	PMCA (Forward)	AF236669	GGCTGTCACCATCTCCTTGGCTTATT	138 bp

	PMCA (Reverse)		TTGGTGGTTAGCGTGCCTG TCTT	
	RPL7 (Forward)	XM_0034 43469	TGAAGGCCATGCGTGTCAA GAA	108 bp
	RPL7 (Reverse)		TCTGCCTGTACTCCTTGTGG TACTT	
Goldfish	*NCX1b* (Forward)	JZ977501	AGATGCTGAACTCTCACGC AG	100 bp
	NCX1b (Reverse)		GCCATTTCACGTCTTGCTTC CT	
	ECaC (Forward)	JZ977502	TGGTCCCAGACTCCACTGA A	148 bp
	ECaC (Reverse)		TGCAGATCTGGACCCTGCT A	
	PMCA (Forward)	JZ977503	TGTGGTGCGCAATGGAAAC G	147 bp
	PMCA (Reverse)		GTTAAAGCGCTCTCGTCGA TTTT	
	18S rRNA (Forward)	MH29580 8	AAACGGCTACCACATCCAA G	166 bp
	18S rRNA (Reverse)		CACCAGATTTGCCCTCCA	
