# Supplementary material for: Comparison of Calcium Balancing Strategies During Hypothermic Acclimation of Tilapia (Oreochromis mossambicus) and Goldfish (Carassius auratus)
Source: Front Physiol. 2018 Sep 3;9:1224. doi: 10.3389/fphys.2018.01224 (PMC6129941; doi:10.3389/fphys.2018.01224)
Supplement: Supplementary file 2 [file Data_Sheet_2.PDF]

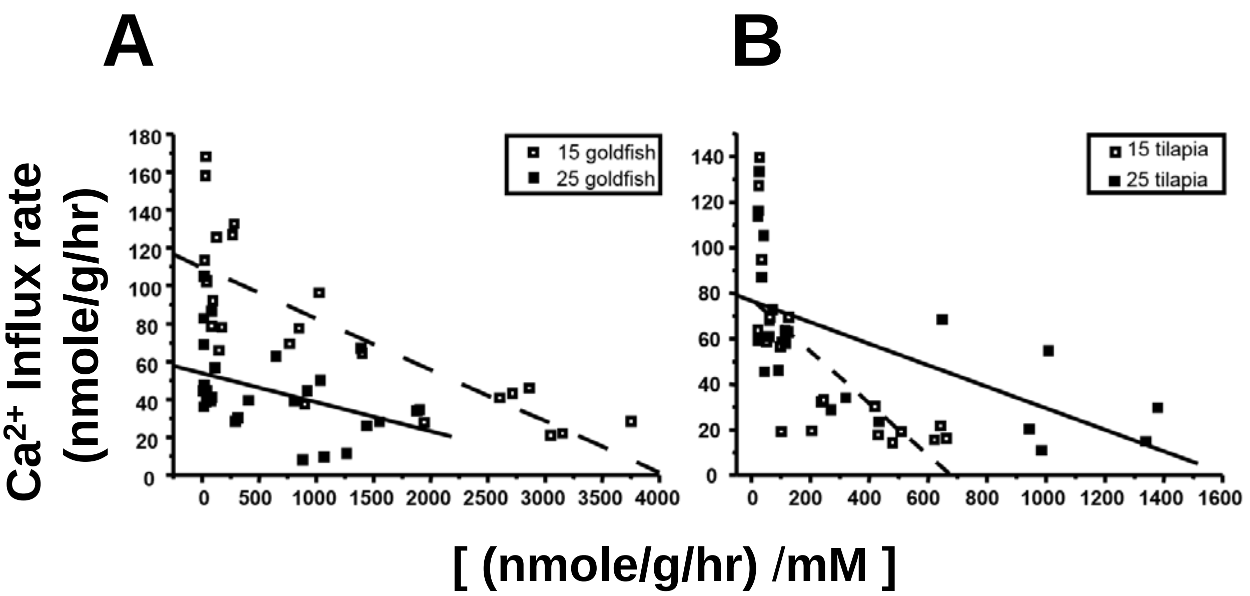

Figure S1. Eadie-Hofstee analysis of Ca^{2+} influx rates. Ca^{2+} influx rates were determined in goldfish (A) and tilapia (B) that were acclimated to 25˚C (solid squares) or 15˚C (open squares). Fishes were incubated with different Ca^{2+} concentrations, ranging from 0.01 to 5 mM with a constant Ca^{2+}/ $^{45}Ca^{2+}$ ratio. Ca^{2+} influx rates were plotted against Ca^{2+} influx rates/Ca^{2+} concentration. Each point indicates a single fish. Liner regression was performed on each experimental group. V_{max} is the Y-axis intercept and the slope is -K_m.
