# Supplementary material for: Comparison of Calcium Balancing Strategies During Hypothermic Acclimation of Tilapia (Oreochromis mossambicus) and Goldfish (Carassius auratus)
Source: Front Physiol. 2018 Sep 3;9:1224. doi: 10.3389/fphys.2018.01224 (PMC6129941; doi:10.3389/fphys.2018.01224)
Supplement: Supplementary file 3 [file Data_Sheet_3.PDF]

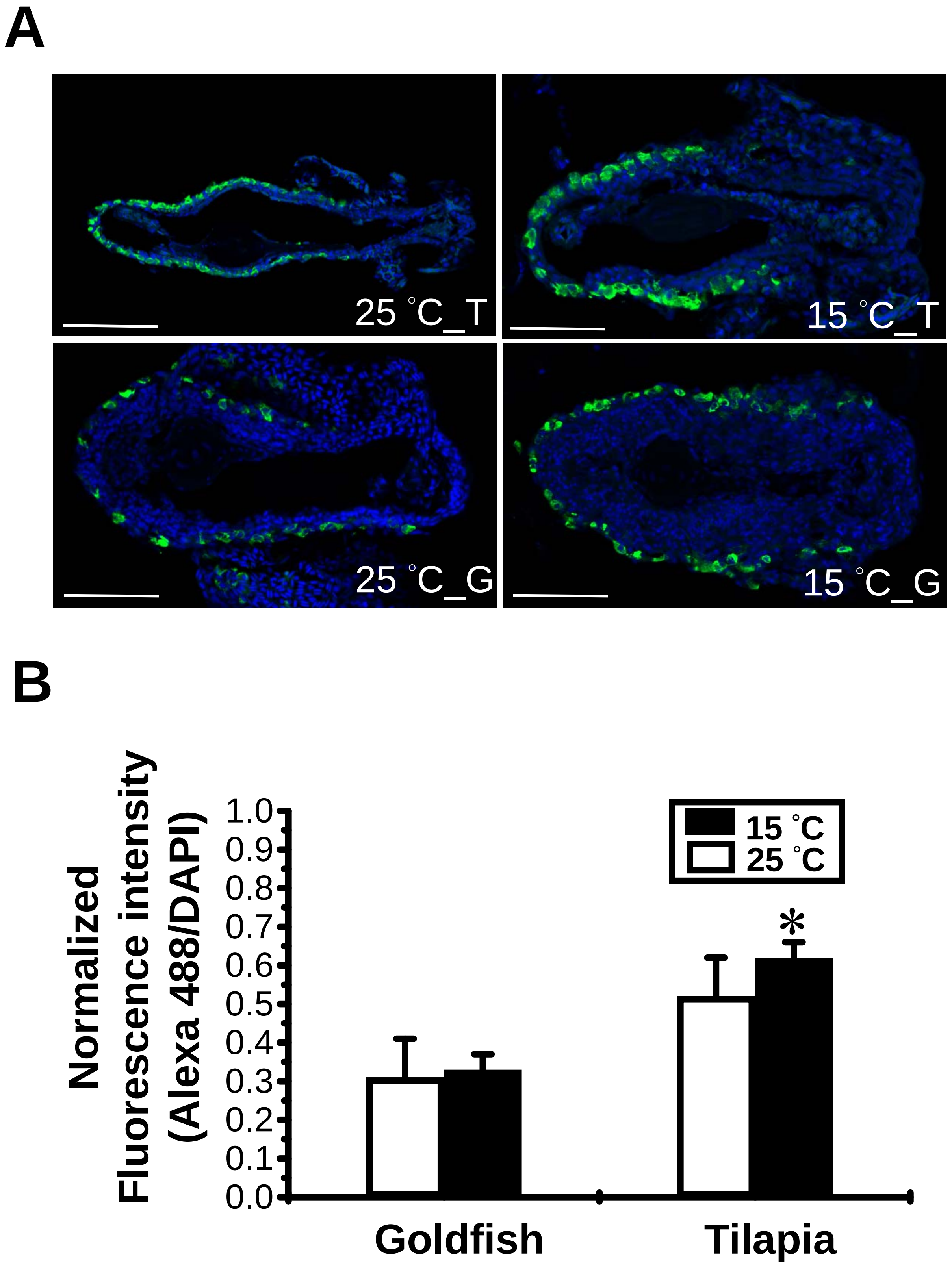

Figure S2. Fluorescence intensity of Na^+/K^+-ATPase immunostaining. (A) Na^+/K^+-ATPase on 25°C (left column) or 15°C acclimated (right column) tilapia (first row) and goldfish (second row) gill sections were immunostained and counterstained with DAPI. Scale bar, 100 µm. Fluorescence was normalized to DAPI (Alexa 488/DAPI). The intensities of three sections from different gill filaments of each fish were measured. Data are expressed as mean ± S.D (three fish in each group, n = 3). The means of control temperature (25°C) and cold-acclimated (15°C) groups from the same species were compared by Student's t-test, $*p \leq 0.05$.
