# Supplementary material for: Comparison of Calcium Balancing Strategies During Hypothermic Acclimation of Tilapia (Oreochromis mossambicus) and Goldfish (Carassius auratus)
Source: Front Physiol. 2018 Sep 3;9:1224. doi: 10.3389/fphys.2018.01224 (PMC6129941; doi:10.3389/fphys.2018.01224)
Supplement: Supplementary file 4 [file Data_Sheet_4.PDF]

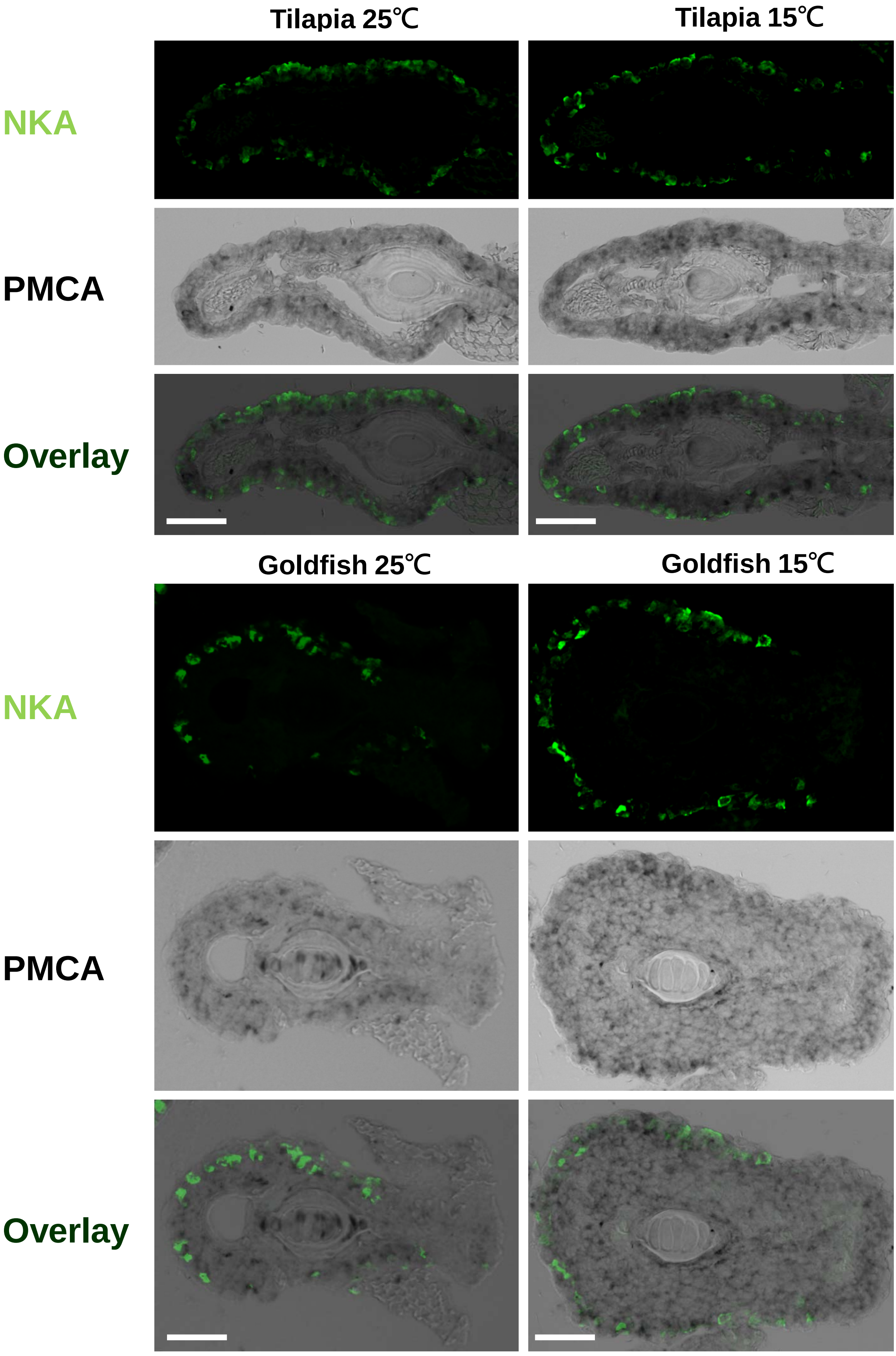

Figure S3. Effect of cold acclimation on the Na^+/K^+-ATPase and PMCA-expressing ionocytes (Original pictures of Figure 6A). Scale bar, 20 µm.
